# Meniscal repair failure following concurrent primary anterior cruciate ligament reconstruction: results from the New Zealand ACL Registry

**DOI:** 10.1007/s00167-023-07424-w

**Published:** 2023-05-05

**Authors:** Richard Rahardja, Hamish Love, Mark G. Clatworthy, Simon W. Young

**Affiliations:** 1 Department of Surgery, Faculty of Medical and Health Sciences, University of Auckland, Auckland, New Zealand; 2 Forte Sports, Christchurch, New Zealand; 3 Department of Orthopaedic Surgery, Middlemore Hospital, Auckland, New Zealand; 4 Department of Orthopaedic Surgery, North Shore Hospital, Auckland, New Zealand

**Keywords:** Anterior cruciate ligament, ACL reconstruction, Meniscal tears, Meniscal repair, Meniscectomy

## Abstract

**Purpose:**

This study aimed to identify the risk factors for meniscal repair failure following concurrent primary anterior cruciate ligament (ACL) reconstruction.

**Methods:**

Prospective data recorded by the New Zealand ACL Registry and the Accident Compensation Corporation were reviewed. Meniscal repairs performed during concurrent primary ACL reconstruction were included. Repair failure was defined as a subsequent reoperation involving meniscectomy of the repaired meniscus. Multivariate survival analysis was performed to identify the risk factors for failure.

**Results:**

A total of 3,024 meniscal repairs were analysed with an overall failure rate of 6.6% (n = 201) at a mean follow-up of 2.9 years (SD 1.5). The risk of medial meniscal repair failure was higher with hamstring tendon autografts (adjusted HR [aHR] = 2.20, 95% CI 1.36–3.56, p = 0.001), patients aged 21–30 years (aHR = 1.60, 95% CI 1.30–2.48, p = 0.037) and in patients with cartilage injury in the medial compartment (aHR = 1.75, 95% CI 1.23–2.48, p = 0.002). The risk of lateral meniscal repair failure was higher in patients aged ≤ 20 years (aHR = 2.79, 95% CI 1.17–6.67, p = 0.021), when the procedure was performed by a low case volume surgeon (aHR = 1.84, 95% CI 1.08–3.13, p = 0.026) and when a transtibial technique was used to drill the femoral graft tunnel (aHR = 2.30, 95% CI 1.03–5.15, p = 0.042).

**Conclusion:**

The use of a hamstring tendon autograft, younger age and the presence of medial compartment cartilage injury are risk factors for medial meniscal repair failure, whereas younger age, low surgeon volume and a transtibial drilling technique are risk factors for lateral meniscal repair failure.

**Level of evidence:**

Level II.

## Introduction

Rupture of the anterior cruciate ligament (ACL) is a devastating injury for athletes and is often accompanied by injury to the menisci in up to 80% of patients [2, 9, 17, 27, 30, 33, 36, 41]. When reconstructing the ACL, meniscal injuries may be managed by resection, repair or no treatment, depending on the location, stability and extent of injury. Success rates of meniscal repair vary considerably, with up to 44% of patients undergoing subsequent meniscectomy for reasons including a nonhealing repair or new trauma [13, 15, 22, 29, 32, 34]. To reduce the risk of subsequent failure of meniscal repair, it is important to establish the rates of failure, as well as the patient and surgical factors that increase the risk of failure.

Studies from national ACL reconstruction registries have risen in prominence due to the availability of large patient populations and the ability to provide direct feedback to surgeons [6]. In addition to ACL reconstruction data, the New Zealand ACL Registry collects data on the presence of meniscal injury at the time of surgery and the type of meniscal treatment undertaken [24]. Following surgery, patient outcomes are collected by the New Zealand ACL Registry and cross-referenced with the Accident Compensation Corporation (ACC) claims data. The ACC is the Government funder of nearly all ACL reconstructions performed in New Zealand, as well as any related subsequent reoperations [37]. This allows for accurate capture of any patient undergoing subsequent meniscectomy following a meniscal repair in concurrent ACL reconstruction.

This study aimed to combine data recorded from the New Zealand ACL Registry with the ACC reoperations dataset to identify the rate and risk factors for meniscal repair failure following concurrent primary ACL reconstruction. It was hypothesised that medial repairs would have a higher failure rate when compared to lateral repairs with different patient and surgical factors influencing the risk of meniscal repair failure. The results of this study will provide feedback to surgeons of any techniques that may increase meniscal repair failure during concurrent ACL reconstruction and may inform patients of their risk of requiring further surgery.

## Materials and methods

This study and collaboration was approved by the ACC Research Ethics Committee. All patients recorded in the registry have signed consent forms for their data to be used and shared between the New Zealand ACL Registry and ACC for the purpose of research. Lastly, the operation of the registry has been declared as a protected quality assurance activity by the New Zealand Government’s Ministry of Health.

### The New Zealand ACL Registry

The New Zealand ACL Registry is a nation-wide registry that was established in 2014 to prospectively capture data on patient, surgical and follow-up variables. Since 2017, it is mandatory for all orthopaedic surgeons who perform ACL reconstructions to actively participate in the registry in order to achieve recertification [46]. As of 2018, based on comparisons to Government healthcare data, it is estimated that approximately 85% of all ACL reconstructions performed in New Zealand are captured by the registry [47].

Patient demographic data is collected through a pre-operative patient questionnaire. An operative data form detailing each reconstruction procedure is completed by the surgeon. This includes the presence of any injury to the menisci and any subsequent meniscal surgery (resection or repair) performed. Subsequently, patients and surgeons can report post-operative complications, including reoperations, and this is recorded in the Registry database. Any reoperation reported to the registry is reviewed by the Registry Administrator who contacts the operating surgeon for the operation note. The operation note is manually reviewed with details of the procedure retrieved and documented in the Registry database.

### Accident Compensation Corporation (ACC)

The ACC is the New Zealand Government’s sole provider of accident insurance for all injuries. As nearly all ACL injuries are accidental, patients who undergo ACL reconstruction are fully funded. Any subsequent treatment related to the reconstruction, such as a reoperation, is also funded. Every procedure funded by the ACC is recorded in a database which includes other details such as the injury mechanism, date, side, location of injury as well as the cost of treatment. Although the ACC lacks the in-depth intraoperative data that is recorded by the New Zealand ACL Registry, it is able to identify whether a patient who has undergone an ACL reconstruction has had any subsequent reoperations.

### Patient population and inclusion criteria

Meniscal repairs performed during a primary isolated single-bundle ACL reconstruction were retrospectively reviewed. The study period was April 2014 to May 2020, allowing for a minimum follow-up of six months. Patients who underwent multi-ligament reconstruction, osteotomy or unicompartmental knee replacement were excluded. Meniscal repairs performed for root tears were excluded (n = 24 medial root tears and n = 134 lateral root tears).

### Outcome of interest

The primary outcome of interest was meniscal repair failure, defined as a repair that underwent subsequent meniscectomy. In this study, a reoperation for meniscectomy was recorded in two ways:

1. Reoperations reported to the Registry through the post-operative complication form
2. Reoperations recorded by the ACC database

In New Zealand, every patient who uses healthcare services has a National Health Index (NHI) number that allows them to be uniquely identified within the healthcare system. In this study, the NHI of every patient was used to match their records from the Registry to the ACC database. Any reoperation that was recorded in the ACC database was retrieved. For every reoperation identified, the operation note was reviewed by the Registry Administrator. The exact details of the reoperation were retrieved, including any subsequent meniscectomy and the side (medial versus lateral). There were five cases where both menisci were repaired and underwent a subsequent meniscectomy but the side(s) that was resected was not specified on the reoperation note. In these cases, we included this as a failure of both repairs.

### Predictor variables

The predictor variables of interest were recorded by the New Zealand ACL Registry through the pre-operative patient questionnaire and the operative data form completed by the surgeon. This included patient age, sex, time from injury-to-surgery, history of previous knee surgery, examination under anaesthesia findings, graft type, graft diameter, femoral tunnel drilling technique, the location and degree of cartilage injury, graded according to the International Cartilage Injury Repair Society (ICRS) scale, and any treatment of a cartilage injury. Meniscal repair technique, implant or suture choice was also recorded. Surgeon volume was calculated as the average number of primary ACL reconstructions recorded annually in the Registry.

### Statistical analyses

Descriptive statistics were provided as mean values with standard deviation (SD) or median values with interquartile ranges (IQR). Continuous variables were assessed for normality through visualisation of Q-Q plots and histograms. Univariate analysis of the rate of meniscal repair failure was performed using Chi-Square Test for categorical variables and Student *t*-test or Mann–Whitney U Test for continuous variables. Multivariate survival analysis of the risk of meniscal repair failure was performed via a Cox proportional hazards regression model. The assumption of proportional hazards was assessed via log(-log) plots. Hazard ratios (HR) with 95% confidence intervals (CI) were computed to identify independent risk factors for meniscal repair failure. Results were considered statistically significant at p < 0.05. All analyses were performed using IBM SPSS Statistics version 25.

## Results

Between April 2014 and May 2020, a total of 6125 out of 10,288 primary ACL reconstructions (60%) involved concurrent meniscal surgery (resection or repair) of either the medial or lateral meniscus. Patient demographics are described in Table 1. Concurrent surgery was performed in both menisci in 1381 patients (13%). There were 3024 meniscal repairs performed in 2699 patients, of which 1814 were a medial repair (60%) and 1210 were a lateral repair (40%). Both menisci were repaired in 325 patients (12%). Of the 1814 medial repairs, 1640 were performed using an all inside suture device (90%), 159 using an outside in or inside out suture (9%) and 15 using both techniques (1%). Of the 1210 lateral repairs, 1074 were performed using an all inside suture device (89%), 125 using an outside in or inside out suture (10%) and 11 using both (1%). The overall failure rate of a meniscal repair was 6.6% (n = 201) at a mean follow-up of 2.9 years (SD ± 1.5 years).

**Table 1:** Baseline demographics of patients with and without concurrent meniscal surgery at the time of the primary ACL reconstruction

Demographic	Total	No Meniscal Surgery		Concurrent Meniscal Surgery	
		n	%	n	%
*Primary ACL Reconstructions*	10,288	4163	40.5%	6125	59.5%
Sex, n (%)					
Male	5868	2198	52.8%	3670	59.9%
Female	4420	1965	47.2%	2455	40.1%
Age (years)					
Continuous					
Mean ± SD	29.1 ± 10.8	28.9 ± 10.3		29.3 ± 11.1	
Categorical, n (%)					
≤ 20	2686	1057	25.4%	1629	26.6%
21–30	3606	1491	35.8%	2115	34.5%
> 30	3996	1615	38.8%	2381	38.9%
Months to Surgery					
Continuous					
Median (IQR)	4.2 (2.6–7.7)	4.1 (2.6–6.9)		4.3 (2.6–8.3)	
Categorical, n (%)					
< 6	6740	2848	68.4%	3892	63.5%
6–12	2080	872	20.9%	1208	19.7%
> 12	1453	437	10.5%	1016	16.6%
NR	15	6	0.1%	9	0.1%
Previous Surgery, n (%)					
Yes	495	195	4.7%	300	4.9%
No	9793	3968	95.3%	5825	95.1%
Graft Choice					
BTB	2412	842	20.2%	1570	25.6%
Hamstring	7304	2975	71.5%	4329	70.7%
NR	572	346	8.3%	226	3.7%
Graft Diameter					
< 8 mm	1449	635	15.3%	814	13.3%
≥ 8 mm	8054	3151	75.7%	4903	80.0%
NR	785	377	9.1%	408	6.7%
Femoral Tunnel Drilling					
Anteromedial Portal	7358	2883	69.3%	4475	73.1%
Transtibial	1168	499	12.0%	669	10.9%
Flipcutter	1028	427	10.3%	601	9.8%
NR	734	354	8.5%	380	6.2%
Surgeon Volume					
< 30 per year	4814	2014	48.4%	2800	45.7%
≥ 30 per year	5474	2149	51.6%	3325	54.3%
Patellar Cartilage, n (%)					
Normal	8307	3369	80.9%	4938	80.6%
Grade 1–4	1571	553	13.3%	1018	16.6%
NR	410	241	5.8%	169	2.8%
Medial Compartment Cartilage, n (%)					
Normal	7195	3255	78.2%	3940	64.3%
Grade 1–4	2733	679	16.3%	2054	33.5%
NR	360	229	5.5%	131	2.1%
Lateral Compartment Cartilage, n (%)					
Normal	8334	3486	83.7%	4848	79.2%
Grade 1–4	1531	430	10.3%	1,101	18.0%
NR	423	247	5.9%	176	2.9%
Cartilage Treatment					
None	9396	3941	94.7%	5,455	89.1%
Chondroplasty	892	222	5.3%	670	10.9%

### Medial meniscal repair failure

One-hundred and forty-two out of 1814 medial meniscal repairs failed and underwent a subsequent meniscectomy (7.8%, Table 2).

**Table 2:** Failure rates of meniscal repair performed in concurrent primary ACL reconstruction

Demographic	Medial meniscal repair				Lateral meniscal repair			
	Number of repairs	Number of failures	% Failed	P Value	Number of repairs	Number of failures	% Failed	P Value
*Total*	1814	142	7.8%		1210	59	4.9%	
Sex, n (%)				0.854				0.352
Male	983	78	7.9%		688	37	5.4%	
Female	831	64	7.7%		522	22	4.2%	
Age (years)								
Continuous								
Mean ± SD	26.3 ± 9.8	25.9 ± 8.8		0.712	25.0 ± 8.9	21.7 ± 6.9		0.005
Categorical, n (%)								
≤ 20	660	49	7.4%	0.361	482	33	6.8%	
21–30	619	56	9.0%		459	20	4.4%	
> 30	535	37	6.9%		269	6	2.2%	
Months to Surgery								
Continuous								
Median (IQR)	3.8 (2.4–6.8)	3.9 (2.2–7.0)		0.77	3.4 (2.2–6.1)	3.7 (1.9–6.7)		0.638
Categorical, n (%)								
< 6	1260	98	7.8%	0.141	895	41	4.6%	0.508
6–12	325	32	9.8%		202	10	5.0%	
> 12	228	12	5.3%		115	8	7.0%	
NR	1	0	0.0%		0	0		
Previous Surgery, n (%)				0.146				0.942
Yes	44	6	13.6%		22	1	4.5%	
No	1,770	136	7.7%		1,188	58	4.9%	
Graft Choice				< 0.001				0.355
BTB	566	23	4.1%		382	15	3.9%	
Hamstring	1171	114	9.7%		796	41	5.2%	
NR	77	5	6.5%		32	3	9.4%	
Graft Diameter				0.118				0.801
< 8 mm	251	26	10.4%		165	8	4.8%	
≥ 8 mm	1458	109	7.5%		975	43	4.4%	
NR	105	7	6.7%		70	8	11.4%	
Femoral Tunnel Drilling				0.096				0.041
Anteromedial Portal	1391	103	7.4%		973	44	4.5%	
Transtibial	136	17	12.5%		64	7	10.9%	
Flipcutter	188	17	9.0%		106	8	7.5%	
NR	99	5	5.1%		67	0	0.0%	
Surgeon Volume				0.011				0.004
< 30 per year	712	70	9.8%		444	32	7.2%	
≥ 30 per year	1,102	72	6.5%		766	27	3.5%	
Patellar Cartilage, n (%)				0.902				0.409
Normal	1588	124	7.8%		1053	54	5.1%	
Grade 1–4 Injury	186	15	8.1%		118	4	3.4%	
NR	40	3	7.5%		39	1	2.6%	
Medial Compartment Cartilage, n (%)				0.001				0.155
Normal	1295	85	6.6%		955	52	5.4%	
Grade 1–4 Injury	487	55	11.3%		223	7	3.1%	
NR	32	2	6.3%		32	0	0.0%	
Lateral Compartment Cartilage, n (%)				0.613				0.76
Normal	1583	123	7.8%		1007	49	4.9%	
Grade 1–4 Injury	193	17	8.8%		166	9	5.4%	
NR	38	2	5.3%		37	1	2.7%	
Cartilage Treatment				0.671				0.239
None	1665	129	7.7%		1122	57	5.1%	
Chondroplasty	149	13	8.7%		88	2	2.3%	

On univariate analysis, a higher failure rate was associated with the hamstring tendon autograft, an ICRS Grade 1–4 injury in the medial compartment, and when the repair was performed by a low volume surgeon who performs < 30 ACL reconstructions per year.

On multivariate survival analysis, failure of a medial meniscal repair was over two times more likely in patients with a hamstring tendon autograft (adjusted HR = 2.20, 95% CI 1.36–3.56, p = 0.001), 1.6 times more likely in patients aged 21–30 years (adjusted HR = 1.60, 95% CI 1.30–2.48, p = 0.037) and 1.8 times more likely in patients with cartilage injury in the medial compartment (adjusted HR = 1.75, 95% CI 1.23–2.48, p = 0.002, Table 3). Surgeon volume was not a risk factor for medial repair failure.

**Table 3:** Multivariate analysis – Predictors of medial meniscal repair failure

Factor	HR (95% CI)	P Value
Sex		
Male	1.03 (0.73–145)	n.s
Female	Reference	
Age		
≤ 20	1.36 (0.86–2.14)	n.s
21–30	1.60 (1.30–2.48)	0.037
> 30	Reference	
Graft Choice		
BTB	Reference	
Hamstring	2.20 (1.36–3.56)	0.001
Medial Compartment		
Normal	Reference	
Grade 1–4 Injury	1.75 (1.23–2.48)	0.002
Surgeon Volume		
< 30 per year	1.19 (0.83–1.69)	n.s
≥ 30 per year	Reference	

### Lateral meniscal repair failure

Fifty-nine out of 1210 lateral meniscal repairs failed and underwent a subsequent meniscectomy (4.9%, Table 2).

On univariate analysis, a higher failure rate was associated with younger age, low volume surgeons and a transtibial technique when drilling the femoral graft tunnel.

On multivariate survival analysis, the risk of failure was nearly three times higher in patients aged ≤ 20 years when compared to patients older than 30 years (adjusted HR = 2.79, 95% CI 1.17–6.67, p = 0.021, Table 4). Low volume surgeons had a 1.8 times higher risk of failure compared to high volume surgeons (adjusted HR = 1.84, 95% CI 1.08–3.13, p = 0.026). The transtibial femoral tunnel drilling technique was over two times more likely to result in a failure when compared to the anteromedial portal technique (adjusted HR = 2.30, 95% CI 1.03 – 5.15, p = 0.042).

**Table 4:** Multivariate analysis – Predictors of lateral meniscal repair failure

Factor	HR (95% CI)	P Value
Sex		
Male	1.44 (0.4–2.44)	n.s
Female	Reference	
Age		
≤ 20	2.79 (1.17–6.67)	0.021
21–30	1.86 (0.75–4.64)	n.s
> 30	Reference	
Surgeon Volume		
< 30 per year	1.84 (1.08–3.13)	0.026
≥ 30 per year	Reference	
Femoral Tunnel Drilling		
Anteromedial Portal	Reference	
Transtibial	2.30 (1.03–5.15)	0.042
Flipcutter	1.25 (0.57–2.75)	n.s

### Other factors

Patient sex, time from injury-to-surgery, a history of previous knee surgery and graft diameter did not influence the rate of meniscal repair failure.

## Discussion

The most important findings of this study are that the use of a hamstring tendon autograft, younger age and concomitant cartilage injury in the medial compartment are risk factors for medial meniscal repair failure, whereas younger age, low surgeon volume and a transtibial femoral tunnel drilling technique are risk factors for lateral meniscal repair failure. The overall meniscal repair failure rate was 6.6%, with medial repairs demonstrating a higher failure rate (7.8%) when compared to lateral repairs (4.9%).

The medial and lateral menisci are commonly injured structures in patients who have ruptured their ACL [2, 8, 9, 41]. In 16,192 ACL reconstructions recorded in the Kaiser Permanente Registry, meniscal injuries were reported in 61% and 53% of primary and revision ACL reconstructions respectively [17]. In New Zealand, a meniscal injury is reported in 60% and 58% of primary and revision ACL reconstructions respectively [25]. Repairing the meniscus can protect against post-traumatic osteoarthritis, improve return to activity and decrease the force on the ACL graft when compared to resection [4, 14, 21, 38, 45]. However, meniscal repair is associated with a higher rate of reoperation compared to resection [22]. In this study, 8% of medial and 5% of lateral meniscal repairs resulted in a subsequent meniscectomy. A similar rate is reported by Toman et al. who performed a study of 77 meniscal repairs in concurrent primary ACL reconstruction, and found that three patients underwent subsequent meniscectomy (4%) [38]. Rodríguez-Roiz et al. analysed 49 amateur athletes who underwent concurrent meniscal repair and primary ACL reconstruction and found that 8% underwent subsequent partial meniscectomy [28]. However, in a systematic review of 95 studies performed by Paxton et al., 148 out of 1044 meniscal repairs performed in primary ACL reconstruction underwent a reoperation (14%) [22].

The present study found a higher failure rate in repairs of the medial meniscus compared to the lateral meniscus which supports the current literature [15, 22, 29, 38]. This may be explained by structural differences between the menisci, as the medial meniscus is securely attached to the medial collateral ligament and tibial plateau and is therefore less mobile and may face higher strain under loading [15, 22, 28]. Interestingly, the use of a hamstring tendon autograft was the most significant risk factor for medial repair failure. The use of the hamstring tendon autograft is associated with a higher risk of residual laxity following ACL reconstruction when compared to the BTB autograft [19]. As the medial meniscus plays an important role as a secondary knee stabilizer that helps control anterior tibial translation and joint laxity [4, 15, 20], any residual laxity following ACL reconstruction may further increase the strain through the medial meniscus and increase the risk of reinjury [15, 22].

The effect of residual laxity on outcomes of meniscal repair may also be demonstrated by the finding of a higher lateral repair failure rate when a transtibial technique was used to drill the femoral graft tunnel when compared to an anteromedial portal technique. When using the transtibial drilling technique, the position of the femoral graft tunnel is limited and restricted to the angle of the tibial tunnel. It is therefore more likely to result in a vertical graft orientation and a non-anatomical ACL reconstruction [39]. As a result, the transtibial technique may lead to greater residual laxity and rotational instability which may explain the higher rate of meniscal repair failure [1, 12, 35].

In the present study, younger age was associated with a higher risk of both medial and lateral meniscal repair failure. Younger age is also the most commonly reported risk factor for ACL graft rupture [7, 11, 16, 25, 26, 44]. The association between younger age and repeat injury is likely to be related to their return to activity as younger patients are more likely to return to high-contact pivoting sports [5, 42–44]. Other possible explanations are related to low adherence to rehabilitation protocols and premature return to activity in young athletes [15, 23, 40].

Surgeon volume is a commonly analysed variable in outcomes of knee surgery, with some studies suggesting poorer outcomes in low volume surgeons [3, 10, 18]. In a study of 77,899 ACL reconstructions performed in New York State hospitals, a 29% decreased risk of ipsilateral knee surgery was reported in procedures performed by surgeons with a volume of > 35 cases per year [31]. Lyman et al. performed a study of 9,609 meniscal repairs and found that a surgeon volume of ≥ 24 cases a year decreased the risk of failure in isolated repairs, but not in concurrent ACL reconstruction [15]. In contrast, our study of meniscal repair in concurrent ACL reconstruction found an almost two times higher risk of lateral meniscal repair failure when the procedure was performed by a surgeon with an average volume of < 30 cases per year, but no difference in medial meniscal repair failure rates. Interestingly, this study demonstrated that nearly 50% of primary ACL reconstructions are being performed by surgeons who do less than 30 reconstructions a year. These findings support previous literature suggesting higher volume surgeons have lower reoperation rates following ACL reconstruction with concurrent meniscal repair [15].

This study is limited to analysing the rate of subsequent meniscectomy as a proxy measure for repair failure. As not all patients who reinjure their meniscus will proceed to a second surgical procedure, this will underestimate the true rate of repair failure. However, subsequent meniscectomy is the standard outcome measure that is most frequently used in the literature to define a meniscal repair failure [15, 22]. Using this outcome therefore allows for comparisons with other studies and may assist future meta-analyses. Another strength of the present study was the combination of data from two national databases, the New Zealand ACL Registry and the ACC. The operation note for each reoperation was manually reviewed which ensured accurate identification of whether a meniscectomy was performed and the side of the meniscus (medial or lateral) that was resected. Another limitation of this study is the mean follow-up of three years, which therefore represents early results. However, this study focused on analysing surgical risk factors which are likely to contribute to early rather than late failures. Furthermore, the Cox proportional hazards regression model was used to adjust for differences in follow-up between patients. Lastly, details related to size and classification of the meniscal tear, the number of sutures implanted when repairing the meniscus and differences in rehabilitation protocol are not recorded by the Registry, and are limitations of such retrospective analyses. Although Registry studies can offer large patient numbers and demonstrate associations, they are unable to investigate the cause of the association and do not infer causality. Future prospective studies should aim to analyse these factors.

The clinical relevance of this study is that surgeons should be aware of the effect of graft choice when repairing a meniscal tear during concurrent ACL reconstruction. Patients can be advised of the higher failure rate of medial versus lateral repairs, as well as the effect of patient age, concomitant cartilage injury and surgeon case volume on meniscal repair outcomes.

## Conclusion

In over 3000 meniscal repairs performed concurrently with primary ACL reconstruction at a mean follow-up of 3 years, the overall rate of subsequent meniscectomy was 6.6%. The use of hamstring tendon autografts, younger age and concomitant cartilage injury in the medial compartment were associated with medial meniscal repair failure. Younger age, surgeons who performed an annual average case volume of less than 30 primary ACL reconstructions and a transtibial femoral tunnel drilling technique were risk factors for lateral meniscal repair failure.

## Data Availability

Data not available.
